# Elaboration of the Al-Al_3_Ni Alloy Eutectic by a Carbothermal Process

**DOI:** 10.1155/2022/7764487

**Published:** 2022-10-26

**Authors:** Imane Bahaj, Mohammed Kaddami, Mohamed Essahli

**Affiliations:** ^1^Hassan First University of Settat, Faculty of Science and Technology, Laboratory of Research Physical-Chemistry of Processes and Materials, Settat 26000, Morocco; ^2^Hassan First University of Settat, Faculty of Science and Technology, Laboratory of Research Applied Chemistry and Environment, Settat 26000, Morocco

## Abstract

The present study develops an elaborate method of materials Al-Al_3_Ni by carbothermic reduction of nickel oxide from the decomposition of Ni(NO_3_)_2_.4H_2_O mixed with aluminum powder. This nitrate salt represents the source of nickel for an elaboration of these materials. The thermodynamic parameters adopted in the experimental study were defined based on the liquid-solid phase equilibrium diagram of the binary system Al-Ni and the Ellingham approach. Throughout this study, the metal Ni is elaborated by carbothermic reduction of nickel oxide resulting from nitrate salt decomposition at 900°C under a nitrogen atmosphere. The heat treatment at 900 °C of a mixture of nitrate salt with aluminum powder under an inert gas atmosphere leads to the formation of phases Al_2_O_3_, Al_4_C_3_, Al, and Ni. The annealing of these obtained phases during 1 h and under a nitrogen flow atmosphere at the temperature of 600°C allowed us to obtain the Al-Al_3_Ni alloy devoid of oxides. The characterization of these obtained solid phases was carried out by the XRD analysis, the SEM-EDS, the DTA, and the DSC.

## 1. Introduction

In the last few years, Metal matrix composites (MMC) technology has become very popular. Particularly, aluminum alloys have the potential for applications in various fields due to their low density (2.7), and high corrosion resistance, also their excellent thermal and electrical conductivity [1–3]. Among these alloys, we found the intermetallic compounds (IMCs) of the Al-Ni system, which are widely used in aircraft construction, automotive engines, fuel cell technology, electricity generation, and energy conversion equipment as well as good candidate materials for coating at high temperature [4]. These intermetallic appear as promising materials for the reinforcement of aluminum alloys. They are visible in the reaction zone between particle and matrix (interface) which leads to a continuous bond between reinforcement and matrix, inexistent in ceramic-reinforced metal matrix composites (SiC, Al_2_O_3_,… etc.) [5]. In particular, the aluminum matrix composites (AMC) reinforced by the intermetallic compounds Al_3_Ni in-situ have received considerable attention in the automotive industry, and it is one of the most important intermetallic based-aluminum [5–8], due to their excellent properties: its high hardness (841 HV), its great wear resistance (2160 MPa) [5, 7, 9], its low coefficient of thermal expansion [6, 7, 10, 11], and its thermodynamic stability [9].

A large variety of methods were reported for the aluminum alloys Al-Al_3_Ni involving casting [6, 12–25], solidification [26–29], arc melting [28], combustion method [27], aluminothermic reduction [30], mechanical alloying [31], and high-pressure torsion (HPT) [32]. Each of these methods has its advantages depending on the field of application. However, many techniques are relatively complicated or expensive [26]. Some of these methods pose some problems, for example, the pyrometallurgical aluminothermic processes take place at high temperatures [30], the mechanical alloying requires a long processing time, the combustion method produces porous materials, and during melting, the casting process causes segregation and alloy evaporation [33].

The purpose of this work is the development of another elaboration method of alloys Al-Al_3_Ni allowing the elaboration of Al-Al_3_Ni alloys based on a carbothermic reduction of nickel nitrate salt “Ni(NO_3_)_2_·4H_2_O” mixed with aluminum powder. This method involves a relatively low-cost reducing agent and it presents good repeatability for industrial applications. In the atmosphere, carbothermic reduction requires a high enough temperature. However, the work under an inert atmosphere significantly reduces the cost of this operation.

This method based primarily on operating the phase equilibria of the binary system Al-Ni [34] is necessary to define the thermodynamic conditions and the stability domains of the intended phases. The implementation of the Ellingham approach [35] provides to define the reduction temperatures of the oxides in the reaction medium under an inert atmosphere by avoiding the oxidation of the elaborated metals.

## 2. Literature Review

**Figure 1:**
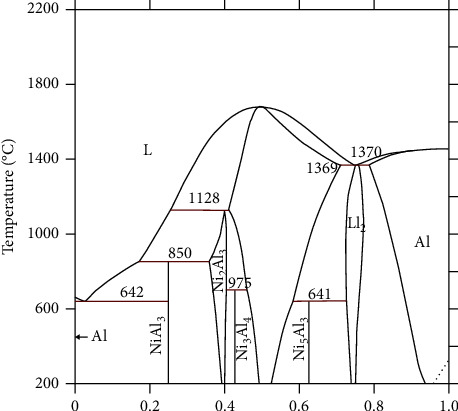
Phase diagram of the Al-Ni binary system [34].

Several researchers have defined the phase equilibrium diagram of the Al-Ni system; recently, the behavior of this system was estimated by the CALPHAD method [34–37]. This diagram includes many mixed products Al_3_Ni, Al_4_Ni_3_, Al_3_Ni_5_, and the solid solutions (Al) (Ni) in the vicinity of the products (Al_3_Ni_2_), (AlNi_3_), and (AlNi) are shown in (Figure 1).

Aluminum (Al) and nickel (Ni) are ductile metals, with both of them having good oxidation strength, aluminum presents a low density [38, 39], and its crystalline structure: face-centered cubic (FCC), with lattice constant *a* = 4.0467 Å, melting point = 660°C and boiling point = 2467°C [39]. Nickel had the same crystalline structure as aluminum with *a* = 3.520 Å [38]. The thermodynamic parameters of nickel and aluminum such as the enthalpy Δ*H*_298_^0^, the free enthalpy Δ*G*_298_^0^, the entropy Δ*S*_298_^0^, and the heat capacity *C*_*P*_ have been estimated by thermodynamic tables [40–43].

The mixed product is of type Fe_3_C [44] rich in aluminum. Its crystallographic structure is orthorhombic with space group: Pnma [44–46]. The lattice parameters of this phase are *a* = 6.698, *b* = 7.352, and *c* = 4.801 [47], and their crystal structure parameters and their lattice constant at equilibrium are well documented by the author Zheng et al. [45]. Al_3_Ni had the most powerful alloy capacity [45]. Moreover, the microstructure of hypoeutectic alloys Al-Ni formed by a dendritic matrix rich in Al (phase *α*) having a eutectic mixture in the interdendritic region composed of *α* and the mixed product Al_3_Ni [13].

This phase exhibits excellent thermal properties in *T* = 500°C due to their good chemical stability [46]. It also shows high cohesive energy, stronger covalent bond [45], good specific strength at high temperatures, and excellent antioxidant properties [12]. This intermetallic was little studied compared to other intermetallic compounds for example AlNi_3_ and AlNi [37].

## 3. The Experimental Study Approach

The present work is interested in the elaboration of the metal ‘Ni' and the mixed product “Al_3_Ni.” For these two products, the metal Ni was obtained from nickel nitrate tetrahydrate Ni(NO_3_)_2_.4H_2_O. The heat treatment of these salts releases nitrogen N_2_ and leads to the formation of nickel oxide “NiO” in temperatures around 225 and 310°C [14].

For the synthesis of mixed product, we opted to use the aluminum powder highly pure “200 mesh” with the nickel nitrate salt.

The Ellingham diagram had exploited for the carbothermic reduction of oxides obtained during the elaboration process used. These diagrams have been introduced by H.J.T. [24]; they provide for predicting the equilibria between the metals and their oxides versus temperature and oxygen pressure, and they allow us to know oxidation-reduction reactions possible thermodynamically between two species. They present the lines of various oxidation reactions. The lower oxides are more stable than the superior oxides due to the standard free enthalpies of their formation which are the more negative [6, 15].

### 3.1. Ellingham Approximation: Thermodynamic Calculations

The formation of oxide M_*x*_O_*y*_ from pure substance M is modeled by equation (1):

(1) $$\begin{array}{c} 2\frac{x}{y}M\left(s\right)+O_{2}\left(g\right)=\frac{2}{y}M_{x}O_{Y}\left(s\right)\text{.} \end{array}$$

The thermodynamic parameters that characterize the reaction (1) join up with the definition of reaction-free enthalpy.

(2) $$\begin{array}{c} \Delta_{r}G_{\left(T\right)}=\Delta_{r}G_{\left(T\right)}^{0}-RTLn\frac{P\left(O_{2}\right)}{P^{0}}\text{.} \end{array}$$

In the standard conditions, the function Δ_*r*_*G*^0^(*T*) is described by the relation (3):

(3) $$\begin{array}{c} \Delta_{r}G_{\left(T\right)}^{0}=\left[\Delta_{r}H_{\left(298\right)}^{0}+\int_{298}^{T}\Delta_{r}C_{p}\;dT\right]-T\left[\Delta_{r}S_{\left(298\right)}^{0}+\int_{298}^{T}\left(\frac{\Delta_{r}Cp}{T}\right)\cdot dT\right]\text{.} \end{array}$$

So, the free enthalpy reaction Δ_*r*_*G*_(*T*)_ is composed of two functions Δ_*r*_*G*_(*T*)_^0^ and *Y*_(*T*)_ whose:

(4) $$\begin{array}{c} \Delta_{r}G_{\left(T\right)}=\Delta_{r}G_{\left(T\right)}^{0}-Y_{\left(T\right)}\text{,} \end{array}$$

with

(5) $$\begin{array}{c} Y_{\left(T\right)}=RTLn\frac{P_{\left(O_{2}\right)}}{P^{0}}\text{.} \end{array}$$

**Figure 2:**
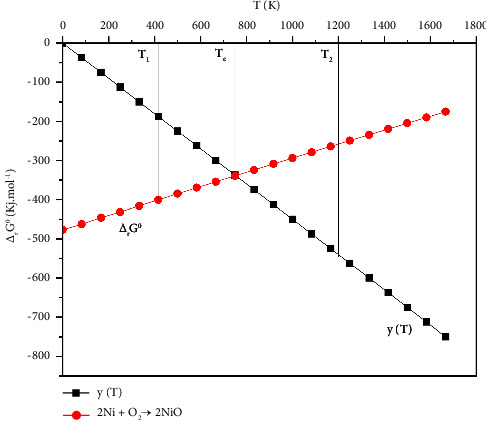
Evolution of the metal-oxide-dioxygen system at fixed oxygen pressure P(O_2_).

At constant oxygen pressure, the straight line *Y*_(*T*)_ passes through the origin of the Ellingham diagram (Figure 2) whose values decrease with increasing temperature. At constant temperature, the values of *Y*_(*T*)_ decrease with the decrease of the oxygen pressure above the reaction medium.

At equilibrium,

(6) $$\begin{array}{c} \Delta_{r}G_{\left(T\right)}=0\text{,}\\ \Delta_{r}G_{\left(T\right)}^{0}=Y_{\left(T\right)eq}\text{.} \end{array}$$

This equilibrium condition, for an oxygen pressure imposed, is reflected in the Ellingham diagram through the intersection of two lines Δ_*r*_*G*_(*T*)_^0^ and *Y*_(*T*)_ at fixed temperature *T*_*e*_ is shown in (Figure 2).

It follows that for an oxygen pressure imposed, and at temperature *T*_1_ lower than *T*_*e*_, the values of *Y*_(*T*)_ will be superior to Δ_*r*_*G*_(*T*)_^0^ and it results that Δ_*r*_*G*_(*T*)_ will be bellow than zero as well the oxide form will be stable. Conversely, for a *T*_2_ superior to a *T*_*e*_, Δ_*r*_*G*_(*T*)_ will be superior to zero, and the reaction shifts for the formation of metal.

A decrease in the oxygen pressure above the reaction medium drops the temperature *T*_*e*_ which favors the stability of the reductants of the reaction (1) at low temperatures.

Based on this principle, the elaboration of our materials has been carried out in the tubular furnace under azote flow for reducing the oxygen pressure of our reactional system and to drop the reduction temperatures of oxides and also to avoid the corrosion of our starting metals and elaborated metals during the carbothermic process.

The principal aim of our work is to produce a metal “Ni” and “Al_3_Ni” thermodynamically stable and exempt from oxides, on the basis of the phase equilibria diagram of the binary system Al-Ni to determine the temperature and system composition for getting the thermodynamic stability of required solid phase, and in the other hand, based on the Ellingham diagram to define the thermodynamic conditions necessary for preventing the oxidation of obtained phases. Hence, the interest is to work under an inert atmosphere for dropping in a maximum oxygen pressure of the reactional system and to avoid the oxidation of phases at modest temperatures. In the end, we will have, spontaneous evolution of the reaction (1) of the system in sense 2 and therefore Δ_*r*_*G*(*T*) > 0 [16].

## 4. Materials and Methods

### 4.1. Elaboration of Materials and Equipment for Heat Treatment

#### 4.1.1. Elaboration of Nickel

All the specimens thermally treated were prepared by the mixture of hydrate Ni(NO_3_)_2_ salt and active carbon with accurately weighed masses. The ensemble was well homogenized in a mortar. However, at the start of our experiments, the nickel nitrate hexahydrate was used as the initial product. Whereas, the hydration degree of this hydrate is higher because it generates the formation of a liquid phase during milling. And as a result, these phenomena incur a remarkable loss of initial products that stay adhered to the mortar surfaces.

In order to prevent the liquid phase formation and to decrease the humidity degree of this hydrate, the thermal decomposition of Ni(NO_3_)_2_.6H_2_O to Ni(NO_3_)_2_.4H_2_O was carried out based on the phase diagram of the binary system Ni(NO_3_)_2_ + H_2_O [17–19]. For that, a quantity of Ni(NO_3_)_2_.6H_2_O had dried in a Proofer (P SELECTA) at the temperature of 130°C for 1 h, the water evaporates leading to the formation of the crystals and their hydration degree is near to the nickel nitrate tetrahydrate at the atmosphere. The hydration rate has been well suitable to make an intimate contact between grains of nickel nitrate obtained and active carbon and to avoid the loss of the raw material and segregation of the mixture during milling.

We put the milled powders in a boat (“Combustion boat, KAV-100”). Then, the sample was placed in a tubular furnace (type LENTON: LTF 16/25/180) completed by a system of thermal treatment under an inert atmosphere “N_2_,” implemented in our laboratory [20, 50] at a temperature of 900°C during 1 h. In the last, the sample was cooled in the furnace under an inert atmosphere to room temperature.

The nickel nitrates are selected in order to elaborate a mixed product at a temperature relatively lower by involving softer chemistry. The choice of carbon resides in its high ability to absorb oxygen, hence its strong utilization as a reducer of metallic oxides.

#### 4.1.2. Elaboration of the Al-Al_3_Ni Alloy

Concerning the synthesis of the mixed product “Al_3_Ni” the rich aluminum in the Al-Al_3_Ni alloy, we operated the same process used for the nickel elaboration. However, the initial samples are composed of the nickel nitrate “Ni(NO_3_)_2_, 4H_2_O,” the active carbon “C” as a reducer, and the aluminum powder “Al.” The quantities of those products and the temperature of thermal treatment were well chosen based on the phase diagram of the binary system Al-Ni (Figure 1).

The temperature of 900°C was fixed for the heat treatments for 1 h in the tubular furnace under inert gas “N_2_.”

Table 1 lists include all the characteristics of the products used in this study:

### 4.2. Characterization Methods

The elaborated solid materials by thermal treatment are subject to milling into homogeneous powders and they are analyzed by using *X*-ray diffraction, which allowed us to define the nature of present solid phases by using the BRUKER : D_2_ Phase (Cu-K*α* 1.541874 A) as the source with an LYNXEYE detector, each pellet was collected for an interval of 2*θ* between 10° and 80° for 20 min.

The microstructure and the morphology of grains are characterized by the scanning electron microscope “SEM” XL30 ESEM PHILIPS equipped with an EDS BRUKER probe.

Phase transformation behavior was studied on the one hand by using differential scanning calorimetry (DSC) coupled with thermal gravimetric analysis (TGA) LABSYS evo simultaneous thermal analyzer. On the other hand, by using the DTG-60H simultaneous DTA-TG Apparatus, the heating rate was 2°C/min from room temperature to 900°C was employed.

## 5. Results and Discussion

### 5.1. The Optimal Thermodynamic Conditions of the Carbothermal Reaction

**Figure 3:**
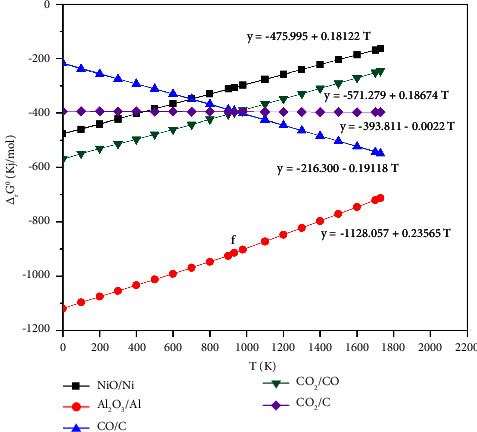
Stability Ellingham diagram of the oxides for the couples NiO/Ni, CO_2_/CO, CO_2_/C, CO/C, and Al_2_O_3_/Al.

The thermodynamic data (standard enthalpy “Δ*H*_298_^0^,” standard entropy “Δ*S*_298_^0^”, and heat capacity “*C*_*p*_” at *T* = 298 K) of oxides and their metals are grouped in Table 2:

Figure 3 shows the Ellingham diagram plot of couples NiO/Ni, CO_2_/CO, CO_2_/C, CO/C, and Al_2_O_3_/Al related to metal oxidation Ni, Al, and C and gas oxidation CO into CO_2_. In this graph, the letter *f* corresponds to the melting point of aluminum at 933 K.

The equations of Δ*G*_*T*_^0^ have been defined for oxidation reactions involving one mole of oxygen O_2_:

(7) $$\begin{array}{c} {2\text{Ni}}_{\left(s\right)}+O_{2\left(g\right)}=2{\text{NiO}}_{\left(s\right)} \end{array}$$

(8) $$\begin{array}{c} {2\text{CO}}_{\left(g\right)}+O_{2\left(g\right)}={2\text{CO}}_{2\left(g\right)} \end{array}$$

(9) $$\begin{array}{c} C_{\left(s\right)}+O_{2\left(g\right)}={2\text{CO}}_{2\left(g\right)} \end{array}$$

(10) $$\begin{array}{c} {2C}_{\left(s\right)}+O_{2\left(g\right)}={2\text{CO}}_{\left(g\right)} \end{array}$$

(11) $$\begin{array}{c} \frac{4}{3}{\text{Al}}_{\left(s\right)}+O_{2\left(g\right)}=\frac{2}{3}{\text{Al}}_{2}O_{3\left(s\right)}. \end{array}$$

According to this diagram, whatever the temperature, the standard free enthalpies lines of couples CO_2_/CO and Al_2_O_3_/Al are placed entirely below the one couple NiO/Ni. As a result, it follows that the carbon monoxide and aluminum can reduce the NiO following the two reactions:

(12) $$\begin{array}{c} {2\text{NiO}}_{\left(s\right)}+{2\text{CO}}_{\left(g\right)}={2\text{Ni}}_{\left(s\right)}+{\text{CO}}_{2\left(g\right)} \end{array}$$

(13) $$\begin{array}{c} {2\text{NiO}}_{\left(s\right)}+\frac{4}{3}\text{Al}\left(s\text{ or liq}\right)={2\text{Ni}}_{\left(s\right)}+{2\text{Al}}_{2}O_{3\left(s\right)}. \end{array}$$

On the one hand, Figure 3 illustrates that the standard free enthalpies line of couple NiO/Ni cuts the couple CO_2_/C at the temperature of 447.18 K, which indicates that the carbon (C) can reduce the NiO at a high temperature of 447.18 K following this reaction:

(14) $$\begin{array}{c} {2\text{NiO}}_{\left(s\right)}+C_{\left(s\right)}={2\text{Ni}}_{\left(s\right)}+C{O_{2}}_{\left(g\right)}. \end{array}$$

On the other hand, the standard free enthalpies line of couple NiO/Ni cuts the couple CO/C at the temperature of 697.75 K; it indicates that the carbon (C) can also reduce the NiO but at high temperature of 697.75 K following this reaction:

(15) $$\begin{array}{c} {2\text{NiO}}_{\left(s\right)}+{2C}_{\left(s\right)}={2\text{Ni}}_{\left(s\right)}+{2\text{CO}}_{\left(g\right)}. \end{array}$$

It follows from the above that the standard free enthalpies line of couple NiO/Ni is higher than the couples CO_2_/C, CO/C, and Al_2_O_3_/Al for the high temperatures of 697.75 K which induce that the nickel oxide can be reduced by the Al, the CO(g) and the C(s) from this temperature.

This reduction temperature of NiO by a carbon remains a theoretical temperature based on purely thermodynamic considerations of the Ellingham approximation. It does not take into account the kinetics considerations that depend on particle sizes of NiO and carbon as well as their intimate contact in the mixture.

The previous works of Yang and McCormick [21] show that the milling significantly reduced the reaction temperature for the carbothermic reduction of NiO from 1350 K for the unmilled sample with the initial sizes of NiO particles are 200 nm, at ∼650 K for samples milled for 12 hours or longer from the particle sizes of NiO achieve 5 nm. Another recent work of the author Bakhshandeh and their collaborators was studying the effect of mechanical activation on the carbothermic reduction of nickel oxide. This study shows throughout the thermogravimetric analysis (TGA) of several samples that the carbothermic reduction of NiO started at ∼800°C in unmilled samples and ∼720°C and milled samples (1 h) whilst after (25 h) of milling, the temperature decreased to about 430°C [22]. The work of Setoudeh and Welham, report that, generally, the more samples are milled together than milled powders separately, the more the reaction rate of carbothermic reduction considerably increases at lower temperatures [23].

As among our starting products figure the hydrate nickel nitrate salt, which induces the thermal decomposition to lead up to nickel oxide, any milling will be difficult to be feasible and for the kinetics considerations cited above, the thermal treatments were performed at 900°C. It is a temperature that largely exceeds the theoretic temperature of 697.75 K defined from the Ellingham diagram.

In the elaboration of Ni from nitrate salts, the reactional system treated at 900°C will be made up of NiO and the activated carbon that reacts with each other to give the nickel. Once the amount of carbon involved will be exhausted, the obtained Ni can react with the oxygen to return to its oxidized state. To avoid this phenomenon, work under an inert atmosphere is imposed. It decreases the corrosion pressure defined in the Ellingham diagram by the intersection of both lines Δ*G*_(*T*)_^0^ and *Y*_(*T*)_ to generate the temperature *T*_*e*_ is shown in (Figure 2). It will allow for the stabilization of nickel in the reduced state at lower temperatures.

In the synthesis at 900°C of mixed product Al_3_Ni, the oxidation of starting raw material aluminum and the nickel produced by the reduction of carbon can be prevented as well with the thermal treatment under an inert atmosphere. It will allow the interaction between the start aluminum and the produced nickel to give the product mixed Al_3_Ni.

### 5.2. Elaboration of Nickel (Ni) from Nickel Nitrate Salt

It follows from the above statements, in the present work, the thermal treatments of starter products have been made under an inert atmosphere (N_2_) at 900°C.

At first, the heat treatment of a mass of hydration degree salt near Ni(NO_3_)_2_.4H_2_O in the tubular furnace under the nitrogen at 900°C for 1 h was achieved.

**Figure 4:**
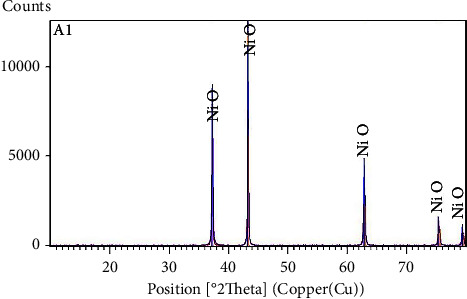
Diffractogram XRD of nickel without reductor.

The characterization by X-ray diffraction of obtaining product led to diffractogram XRD is shown in (Figure 4). A superposition between the experimental XRD diffractogram (red) with the bibliographic data: NiO (JCPDS 01-078-0423-blue) [51].

It confirms that the heat treatment of salts nitrates under the maintained conditions leads well to nickel oxide NiO.

**Figure 5:**
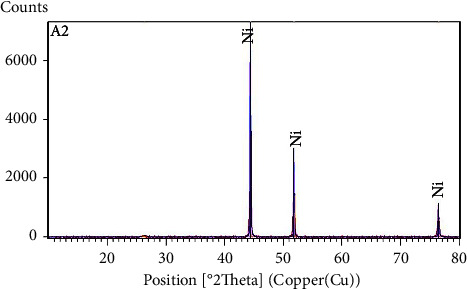
Diffractogram XRD of obtaining nickel by the carbothermic process.

In the second stage, a mass of hydration degree salt near Ni(NO_3_)_2_.4H_2_O was mixed with a carbon amount only necessary to reduce the total mass of NiO deducted from the decomposition of treated nickel hydrate. The whole was milled and was well homogenized in a mortar, then transferred in a boat. This prepared mixture was treated thermally in the tubular furnace at 900°C under a nitrogen atmosphere (N_2_) for 1 h;then, it cools gradually under the nitrogen to room temperature. The characterization by X-ray diffraction of obtaining product led to diffractogram XRD is shown in (Figure 5). A concordance between the experimental XRD diffractogram (red) with the bibliographic data: Ni (JCPDS 01-070-1849-blue) [52].

**Figure 6:**
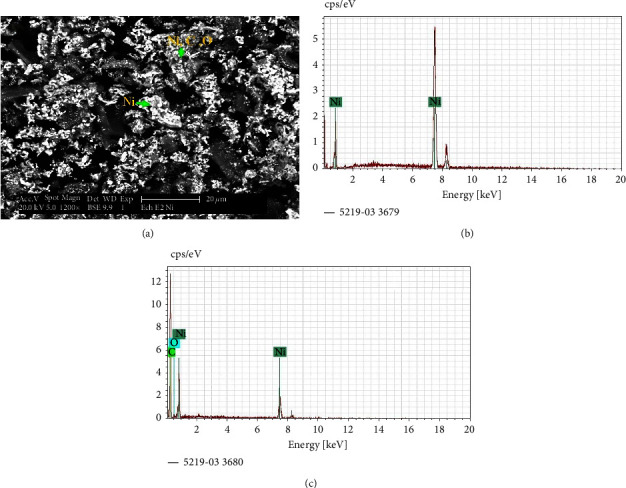
SEM microstructure (in BSE mode) of nickel metal, and typical EDS spectra of this phase.

The characterization by SEM-EDS shown in (Figure 6) confirms the obtainment of nickel by carbothermic reduction of nickel oxides resulting from the heat treatment of nitrate salts.

The grain analysis by EDS shows as well the appearance of fine grains of carbon and nickel oxide not having reacted yet. However, according to the XRD analysis shown in (Figure 5), the amount of this particle remains very low because the relative intensity of their peaks has not been detectable.

The percentages of all constituents are determined by EDS analysis (Figures 6(b) and 6(c)) and are summarized in Tables 3 and 4.

### 5.3. Elaboration of the Mixed Product Al_3_Ni in Equilibrium with Aluminum

**Figure 7:**
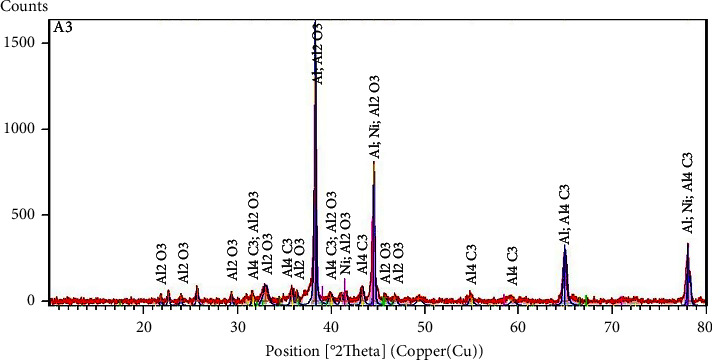
Diffractogram XRD at *T*_1_ = 900°C.

At the start, the mixture was prepared in the stoichiometric ratios 1 Ni(NO_3_)_2_·4H_2_O : 34.5 Al : 3C. The thermal treatment of the start product has been carried out in the tubular furnace under an inert atmosphere (N_2_) at 900°C during 1 h. The characterization by *X*-ray diffraction of obtaining the product led to diffractogram XRD is shown in (Figure 7). The result shows the formation of four phases: Al (JCPDS 01-089-2837 [53]), Ni (JCPDS 00-045-1027 [54]), Al_4_C_3_ (JCPDS 00-035-0799 [55]), and Al_2_O_3_ (JCPDS 01-046-1215 [56]). According to the XRD diffractogram, the relative intensity of elements Ni, Al_2_O_3_, and Al_4_C_3_ is relatively lower than the Al. This reveals that the amounts in the elaborated product are lower.

The analysis XRD of the resultant product from this heat treatment reveals the manifestation of four solid products Al, Ni, Al_2_O_3_, and Al_4_C_3_. The aluminum is a starter product that did not react, the nickel is obtained following the same mechanism described above which is about the decomposition of nitrate salts and then a reduction of solid NiO by the carbon. We can move on that the alumina (Al_2_O_3_) was formed during the preparation of the starting mixture because the oxygen is highly reactive with the aluminum at room temperature. The formation of aluminum carbide Al_4_C_3_ in the produced solid phase involves the alumina being reduced by the solid carbon during this heat treatment.

In 2018, Chahtoua et al. report that in the carbothermic reduction process of alumina (Al_2_O_3_) into aluminum, the addition of aluminum carbide at the start of alumina improves the production yield of aluminum in a very significant way. The yield passes from 1.4% in the case without the additive of Al_4_C_3_ to 21.3% obtained by adding the Al_4_C_3_ additive of aluminum carbide with Al_2_O_3_ : Al_4_C_3_ = 1 : 0.05 in the molar ratio [47]. In the work by Chahtoua et al., the carbothermic reduction reaction of alumina was described by a series of reactions involving the volatile aluminum suboxide Al_2_O (g) obtained during the heat treatment. The formation of Al_4_C_3_ is carried out with the following reactions:

(16) $$\begin{array}{c} {\text{Al}}_{2}O_{3\left(s\right)}+{2C}_{\left(s\right)}={\text{Al}}_{2}O_{\left(g\right)}+{2\text{CO}}_{\left(g\right)} \end{array}$$

(17) $$\begin{array}{c} {2\text{Al}}_{2}O_{\left(g\right)}+{5C}_{\left(s\right)}={\text{Al}}_{4}C_{3\left(s\right)}+{2\text{CO}}_{\left(g\right)}. \end{array}$$

The reaction between the Al_4_C_3_ and the alumina is described by two other reactions involving also the gas phase Al_2_O:

(18) $$\begin{array}{c} {5\text{Al}}_{2}O_{3\left(s\right)}+{2\text{Al}}_{4}C_{3\left(s\right)}={9\text{Al}}_{2}O_{\left(g\right)}+{6\text{CO}}_{\left(g\right)} \end{array}$$

(19) $$\begin{array}{c} {3\text{Al}}_{2}O_{\left(g\right)}+{\text{Al}}_{4}C_{3\left(s\right)}=10{\text{Al}}_{\left(s\right)}+{3\text{CO}}_{\left(g\right)}. \end{array}$$

The aim of our work is the production of mixed product Al_3_Ni. However, for the time of this first heat treatment was revealed insufficient owing to the reaction on the one hand between the alumina and the aluminum carbide Al_4_C_3_ following both equations (18) and (19). On the other hand, according to the Al-Ni binary phase diagram [34], the interaction between the aluminum and the nickel allowing to produce the stable solid Al_3_Ni. From the abovementioned statements, annealing of the production phase for the first heat treatment during 1 h at a temperature 600°C lower than the melting of the aluminum and then of the binary eutectic involving the aluminum solid phases and the Al_3_Ni mixed product were realized.

**Figure 8:**
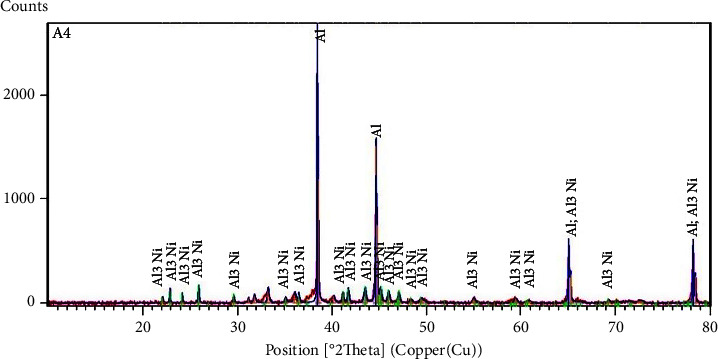
Diffractogram XRD at *T*_2_ = 600°C.

The characterization by *X*-ray diffraction of the obtaining product after annealing leads to diffractogram XRD is presented in (Figure 8). A superposition between the experimental XRD diffractogram (red) and the bibliographic data: Al (JCPDS 01-089-2769-blue) [57] Al_3_Ni (JCPDS 03-065-2418-green) [36] shows the efficiency of annealing in the solid state.

**Figure 9:**
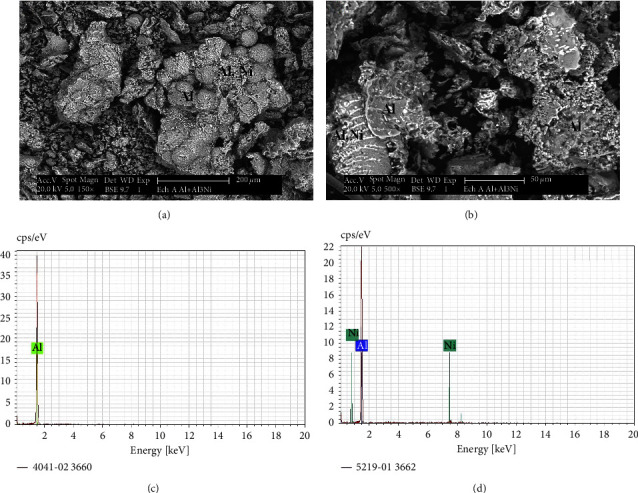
SEM microstructure (in BSE mode) of the Al-Al_3_Ni alloy: (a) 200 *µ*m, (b) 50 *µ*m, and (c) typical EDS spectra of this alloy.

The microstructural characterization by SEM in the BSE mode of the Al-Al_3_Ni alloy after annealing is shown in (Figures 9(a)–9(b)). It perfectly confirms the results from the analysis XRD.

In BSE mode, contrast comparison shows that heavy components backscatter electrons more effectively than light elements [6]. Phase detection is visible, with Ni-rich areas shining brighter than Ni-deficient locations.

There are two microstructure differences in the Al-Al_3_Ni alloy. A white minor contrast of the Al_3_Ni alloy with a dark gray matrix contrast (Al). In the lower part of the left side of (Figure 9(b)), some grains appear as a composite formed by Al_3_Ni fibers of white contrast in the aluminum matrix.

The metal percentages of all constituents of the microstructure are determined by EDS analysis (Figures 9(c) and 9(d)) and are summarized in Tables 5 and 6.

**Figure 10:**
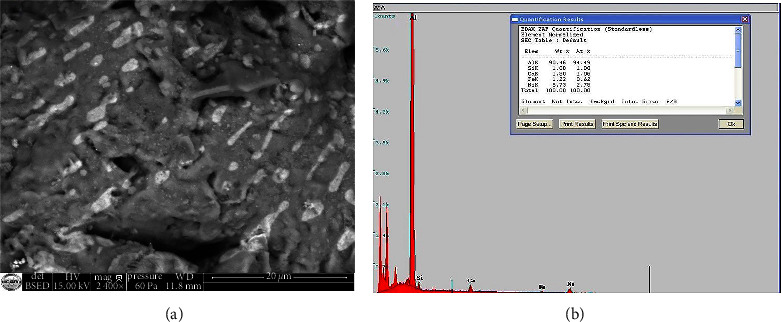
SEM microstructure (in BSE mode) of the Al-Al_3_Ni alloy: (a) (20 *µ*m), (b) typical EDS spectrum of this alloy.

High magnification SEM analysis (Figure 10(a)) revealed traces of some debris from the combustion boat where our alloys underwent heat treatment. In (Figure 10(b)), an EDS spectrum of a region containing this debris is displayed.

### 5.4. Thermal Analysis of the Prepared Al-Al_3_Ni Material

**Figure 11:**
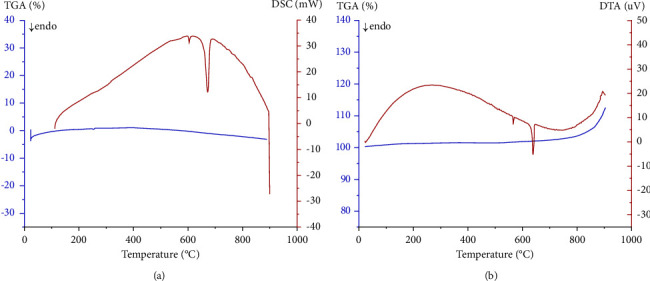
Thermal analysis of the Al-Al_3_Ni alloy. (a) DSC-TGA and (b) DTA-TGA of this alloy.

The thermal analysis of the elaborated material was performed using DSC techniques under an inert atmosphere ‘N_2_' and DTA under an ambient atmosphere for a heating rate of 2°C/min and temperature ranging from 25°C to 900°C as shown in (Figures 11(a) and 11(b)).

Two endothermic peaks were detected. The first less intense peak corresponds to the allotropic transition temperature of the *α*-quartz phase to *β*-quartz at a temperature of 573°C [58–60]. This phenomenon can be confirmed by EDS analysis (Figure 10(b)) which depicts the appearance of some traces of silica from the combustion boat. The second most intense endothermic peak at a temperature *T*_eut_ = 642°C is typical of the eutectic transformation *α* − Al + Al_3_Ni Liquid. This confirms that the alloy elaborated has the eutectic composition of the Al-Ni system [34].

## 6. Conclusions

The present work developed a method for producing Al-Al_3_Ni alloy by carbothermal reduction of nickel oxide from Ni(NO_3_)_2_·4H_2_O decomposition. This method is based, on the one hand, on phase diagrams to specify the stability domain (composition-temperature) of the various products sought. On the other hand, it involves the Ellingham diagrams to optimize the optimal thermodynamic conditions that allow the prevention of the formation of oxides implicated in the studied system.

This method is simpler and less expensive than the other processes used to elaborate this alloy.

The carbothermic reduction of nickel oxide from nickel nitrate allows for the elaboration of the nickel-metal free of oxides.

The elaboration of Al-Al_3_Ni alloys was synthesized in two stages:

The first stage consists of heat treatment for 1 h at 900°C and under a nitrogen atmosphere leads to several phases: Al_2_O_3_, Al_4_C_3_, Al, and Ni. In the second stage, annealing of these obtained phases during 1 h and under an inert atmosphere at the temperature of 600°C allowed us to obtain the Al-Al_3_Ni alloy exempt of oxides.

XRD and SEM-EDS analysis techniques allowed us to identify and characterize the solid phases that occur.

The mixture treated in this work closely matches the eutectic composition of the Al-Ni binary (0.03 At. %). The thermal analysis of DSC and DTA confirmed well that the elaborated alloy corresponds to the eutectic transformation.

The obtained alloy is free of oxides which confirms the effectiveness of the developed method and encourages its application for the elaboration of the intermetallic material Al_3_Ni (0.25 At. % in Ni) which present excellent mechanical properties.

## Figures and Tables

**Table 1:** Characteristics of the starting materials used.

CAS Number	Chemical compound	Formula	Provider	Purity (%)
13478-00-7	Nickel nitrate hexahydrate	Ni(NO_3_)_2_·6H_2_O	Sigma-Aldrich	>97
7429-90-5	Powder aluminum «200 mesh»	Al	Sigma-aldrich	99.9
64365-11-3	Active carbon	C (graphite)	Sigma-aldrich	99
231-783-9	Nitrogen	N_2_	Scomalab	≥99.9

**Table 2:** Thermodynamic data of the metal oxides present.

Elements	*T* (k)	Δ_*f*_ *H*^0^ (kJ/mol)	Δ_*f*_ *S*^0^ (kJ/K·mol)	*C *_*P*_ (kJ/mol)	References
Ni	298	0	0.0298737	0.0259868	[15]
C	298	0	0.00574	0.008518	[15]
Al(s)	298	0	0.02835	0.02431	[41]
Al(l)	933	10.795	0.01154	0.0318	[6, 12–25, 42–40]
O_2_	298	0	0.205036	0.029375	[15]
NiO	298	−239.44	0.037991	0.044309	[48, 49]
CO	298	−110.53	0.197657	0.029141	[49]
CO_2_	298	−393.51	0.213787	0.037135	[49]
Al_2_O_3_	298	−1675.7	0.05092	0.07901	[41]

**Table 3:** Percentage of Ni metal analyzed by EDS.

5219-03 3679						
Element	At. no.	Netto	Mass (%)	Mass norm. (%)	Atom (%)	Abs. error (%) (1 sigma)
Nickel	28	12542	87.37	100.00	100.00	2.51
		Sum	87.37	100.00	100.00	

**Table 4:** Phase assignment and percentage of microstructure constituents.

5219-03 3680						
Element	At. No.	Netto	Mass (%)	Mass norm. (%)	Atom (%)	Abs. error (%) (1 sigma)
Carbon	6	4533	82.37	76.17	91.46	13.16
Oxygen	8	125	4.44	4.10	3.70	2.06
Nickel	28	1793	21.33	19.72	4.85	0.82
		Sum	108.13	100.00	100.00	

**Table 5:** Percentage of the Al matrix analyzed by EDS.

4041-02 3660						
Element	At. no.	Netto	Mass (%)	Mass norm. (%)	Atom (%)	Abs. error (%) (1 sigma)
Aluminum	13	28251	68.33	100.00	100.00	3.34
		Sum	68.33	100.00	100.00	

**Table 6:** Phase assignment, metal concentrations of microstructure constituents of Al_3_Ni alloy after annealing at 600°C.

5219-01 3662						
Element	At. No.	Netto	Mass (%)	Mass norm. (%)	Atom (%)	Abs. error (%) (1 sigma)
Aluminum	13	10098	81.60	92.12	96.22	4.11
Nickel	28	238	6.98	7.88	3.78	0.54
		Sum	88.58	100.00	100.00	

## Data Availability

The data used to support the findings of this study are included within the article and are also available from the corresponding author.
